# *DMPK* 3′ untranslated repeat expansions in unexplained sudden cardiac death in the young

**DOI:** 10.1093/eurheartj/ehag247

**Published:** 2026-04-03

**Authors:** Zoe Ward, Jackson O’Neill, Rachael Stiles, Hui Hui Phua, Stephanie Burcher, Nikki Earle, Andrew Martin, Jia Xin Mok, Jackie Crawford, Jenny Eaton, Saraya Hogan, Lisa Worgan, Belinda Gray, Charlotte Burns, Tom Donoghue, Martin K Stiles, Laura Yeates, Cristin G Print, Ian Hayes, Jonathan R Skinner, Richard D Bagnall, Polona Le Quesne Stabej

**Affiliations:** Department of Molecular Medicine and Pathology, The University of Auckland, Private Bag 92019, Auckland 1142, New Zealand; Precision Medicine Initiative, The University of Auckland, Auckland, New Zealand; Bioinformatics and Molecular Genetics at Centenary Institute, The University of Sydney, Sydney, Australia; Department of Cardiology, Waikato Hospital, Hamilton, New Zealand; Liggins Institute, The University of Auckland, Auckland, New Zealand; Department of Molecular Medicine and Pathology, The University of Auckland, Private Bag 92019, Auckland 1142, New Zealand; Genetic Health Service New Zealand-Northern Hub, Te Toka Tumai, Auckland, New Zealand; Department of Medicine, The University of Auckland, Auckland, New Zealand; Department of Cardiology, Auckland City Hospital, Auckland, New Zealand; Department of Molecular Medicine and Pathology, The University of Auckland, Private Bag 92019, Auckland 1142, New Zealand; Department of Cardiology, Auckland City Hospital, Auckland, New Zealand; Genetic Health Service New Zealand-Northern Hub, Te Toka Tumai, Auckland, New Zealand; Molecular Genetics, LabPLUS, Te Toka Tumai, Auckland, New Zealand; Clinical Genetics Department, Royal Prince Alfred Hospital, Sydney, Australia; Faculty of Medicine and Health, The University of Sydney, Sydney, Australia; Department of Cardiology, Royal Prince Alfred Hospital, Sydney, Australia; Department of Cardiology, Royal Prince Alfred Hospital, Sydney, Australia; Molecular Cardiology at Centenary Institute, The University of Sydney, Sydney, Australia; Department of Cardiology, Wellington Hospital, Wellington, New Zealand; Department of Cardiology, Waikato Hospital, Hamilton, New Zealand; Department of Medicine, The University of Auckland, Auckland, New Zealand; Genomics and Inherited Disease Program, Garvan Institute of Medical Research and University of New South Wales, Sydney, Australia; Department of Molecular Medicine and Pathology, The University of Auckland, Private Bag 92019, Auckland 1142, New Zealand; Precision Medicine Initiative, The University of Auckland, Auckland, New Zealand; The Maurice Wilkins Centre for Molecular Biodiscovery, Auckland, New Zealand; Genetic Health Service New Zealand-Northern Hub, Te Toka Tumai, Auckland, New Zealand; Heart Centre for Children, Sydney Children’s Hospital Network, Sydney, NSW, Australia; Department of Paediatric and Adolescent Medicine, University of Sydney, Sydney, NSW, Australia; Bioinformatics and Molecular Genetics at Centenary Institute, The University of Sydney, Sydney, Australia; Faculty of Medicine and Health, The University of Sydney, Sydney, Australia; Department of Molecular Medicine and Pathology, The University of Auckland, Private Bag 92019, Auckland 1142, New Zealand

**Keywords:** *DMPK*, Sudden cardiac death, Repeat expansion, Genome sequencing, Oxford nanopore technology

## Introduction

Sudden cardiac death (SCD) is the unexpected death of a previously healthy individual due to a known or suspected cardiac cause. In our Australian/New Zealand SCD in the young (1–35 years) cohort, the annual incidence was ∼1.3 per 100 000.^1^ When no cause of SCD is found following comprehensive postmortem investigation, including toxicology and microbiology analysis, the case is classified as unexplained SCD. Causative variants in cardiac genes are identified in up to 27% of young unexplained SCDs, suggesting other causes exist.^1^ Myotonic dystrophy Type 1 (DM1) is an autosomal dominant, multisystemic disorder with a prevalence of 1:2100 births.^2^ The leading cause of mortality in DM1 is respiratory failure (38%), followed by sudden death (15%).^3^ Myotonic dystrophy Type 1 is caused by a CTG-repeat expansion in the 3′-untranslated region (3′UTR) of the *DMPK* gene. The age of onset and phenotype severity roughly correlate with repeat length: with 50–100 repeats associated with mild disease, ∼100–1000 with classic disease, and >1000 with congenital disease.^4^ Expanded [CUG]_n_  *DMPK* transcripts cause mis-splicing of key cardiac transcripts, including *SCN5A.*^5^ Here, we describe three families with unexplained SCD in the young associated with *DMPK* 3’UTR-repeat expansions in the absence of overt neuromuscular features or evident conduction disease.

## Methods

Family-1 was enrolled in an unsolved inherited heart disease project and underwent short- and long-read genome sequencing. Triplet-primed polymerase chain reaction (TP-PCR)^4^ confirmed the *DMPK* 3′UTR-repeat expansion.

Family-2 and Family-3 were identified from an Australian unexplained SCD cohort (*n* = 115, aged 1–35) with negative toxicology and microbiology analysis, who were prospectively recruited (2010–12)^1^ or recruited to the Genetic Heart Diseases Clinic at Royal Prince Alfred Hospital, Sydney (2014–24). Genetic testing and *DMPK* 3′UTR-repeat sizing were performed by genome sequencing (*n* = 58) or exome sequencing plus TP-PCR (*n* = 57) (AmplideX PCR/CE DMPK Kit, Asuragen).

## Results

### Family-1

**Figure 1:**
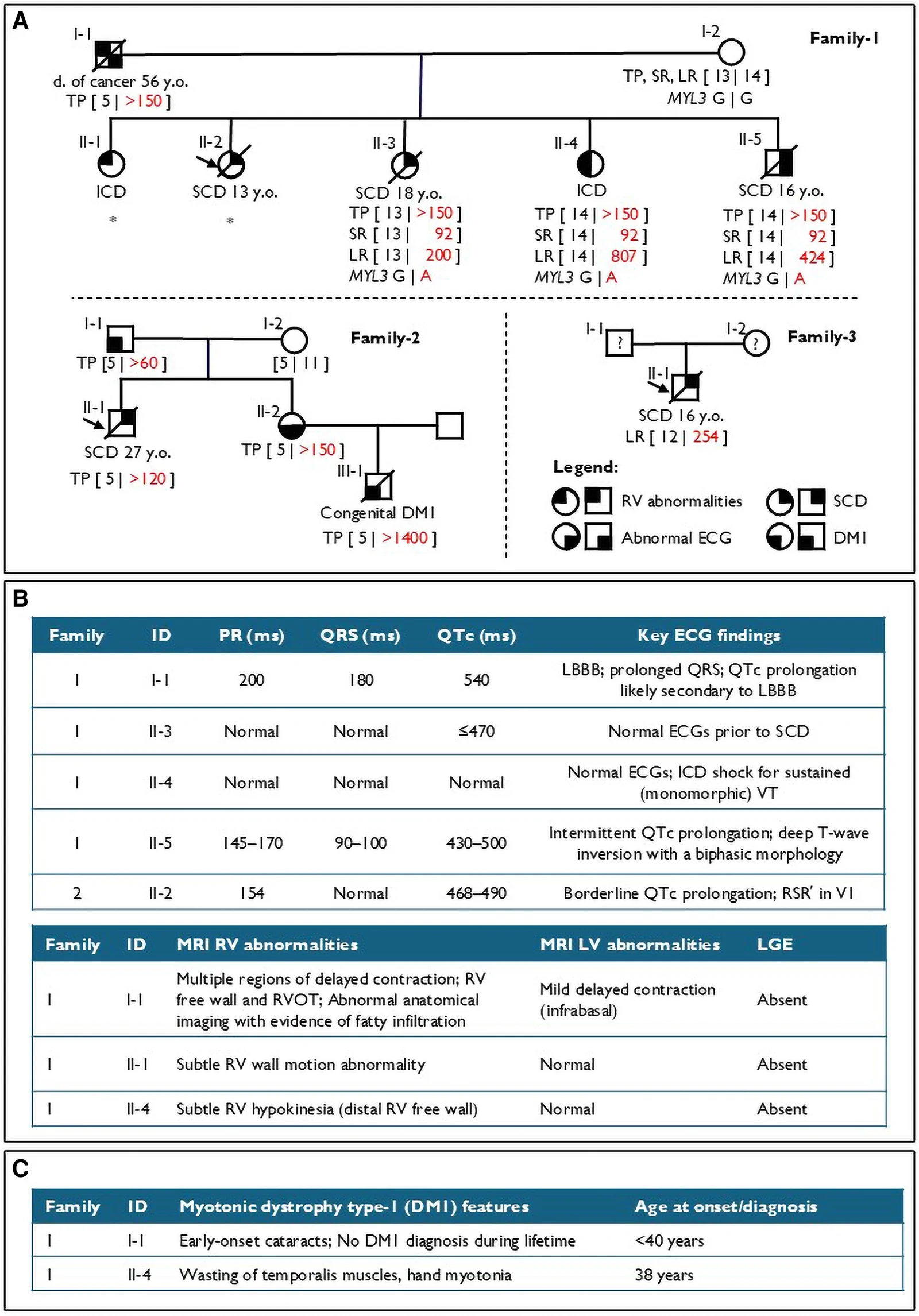
Phenotypical features and *DMPK* [CTG]_n_ 3′-untranslated region-repeat expansion in Family-1–Family-3. *(A*) Pedigrees showing *DMPK* 3′-untranslated region [CTG]_n_ repeat genotypes in Family-1–Family-3 and *MYL3* c.235G > A; p.(Val79Ile), variant of uncertain significance, in Family-1. Red indicates expanded *DMPK* 3′-untranslated region-CTG repeats; black indicates normal range. Family-1: the father’s (I-1) DNA was isolated from formalin-fixed paraffin-embedded tissue and was of insufficient quality to perform long-read sequencing; Family-1 subjects II-1 and II-2, *indicates no DNA available, and subject II-4, DNA was isolated from a peripheral blood sample at age 38, showing somatic expansion of the repeat length with age. Types of sequencing technology are depicted: triplet repeat-primed PCR, short-read sequencing, and long-read sequencing. *MYL3*: NM_000258.3:c.235G > A; p.(Val79Ile): ClinGen classification for *MYL3* indicates a definitive association with hypertrophic cardiomyopathy and a limited association with arrhythmogenic cardiomyopathy. Andersen *et al.*^6^ reported MYL3 p.(Val79Ile) in an hypertrophic cardiomyopathy proband; among nine heterozygous family members, three exhibited borderline hypertrophic cardiomyopathy, while five younger carriers (ages 3–23 years) were asymptomatic. The variant has a minor allele frequency of 0.0088% in gnomAD v4.1.0 and a REVEL score of 0.49 (uncertain). American College of Medical Genetics and Genomics (ACMG) criterion PM2_Supportive is met. Overall classification: variant of uncertain significance. *(B*) Summary of electrocardiogram and cardiac MRI findings: electrocardiogram interpretations: Family-1 (I-1): abnormal electrocardiogram with left bundle branch block, PR-interval upper normal range. Normal-looking repolarization but prolonged corrected QT-interval, which is likely predominantly related to left bundle branch block, causing a prolonged QRS-interval. No advanced atrio-ventricular (AV) block/arrhythmia on Holter. Family-1 (II-3): Serial electrocardiograms showed corrected QT-interval ≤ 470 ms with normal PR-intervals and no evidence of progressive conduction disease. Holter monitoring and exercise testing shortly before sudden cardiac death were normal, with appropriate QT adaptation and no ventricular ectopy. Family-1 (II-4): sinus tachycardia abruptly progressed to likely supraventricular tachycardia, triggering implantable cardioverter-defibrillator shock, followed by 21 beats of tachycardia of indeterminate origin at 300 beats per minute. This was followed by two episodes of non-sustained ventricular tachycardia of seven and 14 beats, respectively. Family-1 (II-5): a single electrocardiogram demonstrated corrected QT-interval prolongation to 500 ms, prompting beta-blocker therapy. Serial electrocardiograms (ages 6–13 years), exercise testing, and Holter monitoring showed stable PR-interval (145–170 ms) and QRS (90–100 ms) intervals, corrected QT-interval of 430–500 ms, normal exercise-related QT adaptation, no ventricular ectopy, and a normal Holter 2 years prior to death. (*C*) Family-1 myotonic dystrophy Type 1 (DM1) phenotype. y.o., year old; d., died; SCD, unexplained sudden cardiac death; DM1, myotonic dystrophy Type 1; ICD, implantable cardioverter-defibrillator; RV, right ventricle; LV, left ventricle; TP, triplet repeat-primed PCR; SR, short-read sequencing (Illumina technology); LR, long-read sequencing (Oxford Nanopore Technology); QTc, corrected QT-interval; LBBB, left bundle branch block; VT, ventricular tachycardia; RVOT, right ventricular outflow tract; LGE, late gadolinium enhancement.

The female proband (II-2) had witnessed SCD during exercise at age 13; autopsy found no cause and a structurally normal 190 g heart. Her father had right ventricular (RV) abnormalities on cardiac magnetic resonance imaging (MRI) and left bundle branch block on electrocardiogram (ECG) (*Figure 1A* and *B*); the mother was clinically normal. Over the next decade, two siblings had witnessed SCD: II-5 during exercise at age 16 and II-3 after an argument at age 18. Autopsies showed normal hearts: II-3, with subtle subendocardial myocyte swelling and scattered contraction bands, suggesting subendocardial ischaemia, and II-5, 398 g heart with widespread contraction bands. Histology: RV not examined in any of the three unexplained SCDs; the left ventricle was normal. Two other siblings (II-1, II-4) received cardioverter-defibrillators. Sudden cardiac death individuals (II-3, II-5) had no conduction disease on ECG, exercise testing, or Holter; II-5 had prolonged corrected QT interval (QTc), and living sibling II-4 had abnormal RV. Long-read genome sequencing identified a heterozygous pathogenic *DMPK* 3′UTR-repeat expansion in II-3, II-5, and II-4. Retrospectively, the family reports that SCD child II-5 had slight clumsiness, and the father had early cataracts (before age 40). II-4 now has DM1 neurological features (*Figure 1C*). A heterozygous, likely paternally inherited *MYL3* (c.235G > A;p.Val79Ile) variant of uncertain significance (VUS) was identified in II-3, II-4, and II-5 (*Figure 1A*) and was previously reported as a low-penetrance variant in a hypertrophic cardiomyopathy (HCM) family.^6^

### Family-2

The male proband (II-1) had witnessed SCD during endurance exercise at age 27. His 604 g heart showed patchy interstitial fibrosis with hypertrophic cardiomyocytes, and no cause of death was determined. Exome sequencing of 59 cardiac genes found no pathogenic variant. His nephew (III-1) died at 4 days with a clinical diagnosis of congenital DM1. Triplet-primed PCR identified a *DMPK* 3′UTR-repeat expansion in the nephew (III-1, >1400 repeats), his mother (II-2, >150), the proband (II-1, >120), and the proband’s father (I-1, >60) (*Figure 1A* and *B*).

### Family-3

The male proband died suddenly at age 16 during exercise. His 405 g heart showed left ventricular lymphocytic inflammation and a septal thickness of 15 mm, and no cause of death was determined. Visual review of exome data revealed two reads with expanded *DMPK* repeats, prompting targeted long-read sequencing of a 185-gene cardiac panel, which confirmed a heterozygous pathogenic *DMPK* 3′UTR-repeat expansion of 254 repeats (*Figure 1A*). Family details were not available.

Family-2 and Family-3 were identified from an Australian cohort of 115 unrelated unexplained SCD with average age 22.2 years (range 1–35 years; 73.9% males), indicating an incidence of 1.7% (Confidence Interval [CI]: 0.3%–4.6%) in this cohort.

## Discussion

We report five unexplained SCDs in the young from three families, in whom initial genetic testing was uninformative. Pathogenic *DMPK* 3′UTR-repeat expansions were later identified using genome sequencing in Family-1, following a neonatal diagnosis of DM1 in Family-2, and via targeted long-read sequencing in Family-3. The *DMPK* 3′UTR-repeat region is not included in cardiac panels, is poorly captured on exome, and is challenging to size expanded repeats using short-read sequencing (*Figure 1A*). Expansions require secondary confirmation with TP-PCR or long-read sequencing. We highlight the value of comprehensive clinical assessment of the proband and extended family to identify clinical clues pointing to the underlying cause of SCD. The *DMPK* 3′UTR-repeat expansions in this study fit the classic range (100–1000).^4^ Although conduction defects are the most common cardiac abnormality in DM1, ventricular tachyarrhythmias also contribute to SCD. In young DM1 patients (10–18 years), tachyarrhythmias are more frequent than conduction disorders and are often exercise triggered.^3,7^ Given the age-related penetrance of classic DM1, young individuals with pathogenic *DMPK* 3′UTR-repeat expansions may lack overt clinical features but remain at risk for SCD. Notably, two children (Family-1) with SCD had no conduction disease on ECG, exercise tolerance testing, or Holter monitoring during life.

Our findings suggest that *DMPK* 3′UTR-repeat expansions should be considered as a potential cause of unexplained SCD in the young. In Family-1, RV histology was not performed on autopsy, and the proband’s DNA was not available; this represents a study limitation. The father had arrhythmogenic cardiomyopathy features and prolonged QTc; two living siblings had abnormal RV.

Among three published young DM1 patients with SCD, one showed mild ECG abnormalities and no cardiac pathology at autopsy, consistent with SCD, while two had RV fibro-fatty infiltration characteristic of arrhythmogenic cardiomyopathy; the effect of other exome-detected variants was unclear.^8^

Whether *DMPK* 3′UTR-repeat expansion alone predicts SCD risk or if other modifiers contribute remains unclear. No additional pathogenic variants were identified in our cases, and the potential role of the *MYL3* variant in Family-1 is uncertain, but *DMPK* 3′UTR-repeat expansions combined with loss-of-function variants in *SCN5A* were reported in two young DM1 individuals with cardiac arrest.^9,10^

Genome and long-read sequencing of larger DM1 and SCD cohorts with detailed phenotypes will clarify repeat characteristics and genetic contributors. We recommend including *DMPK* 3′UTR-repeat sizing in young unexplained-SCD.

## Declarations

### Data Availability

Data supporting the findings of this study are included in the article. Requests for additional data will be considered on a case-by-case basis and may be granted if consistent with the ethical approvals obtained for this study.
